# TNFRSF12A and CD38 Contribute to a Vicious Circle for Chronic Obstructive Pulmonary Disease by Engaging Senescence Pathways

**DOI:** 10.3389/fcell.2020.00330

**Published:** 2020-05-27

**Authors:** Yan Dong, Hongbao Cao, Rongyuan Cao, Ancha Baranova

**Affiliations:** ^1^Lianyungang Second People’s Hospital, Lianyungang, China; ^2^School of Systems Biology, George Mason University, Fairfax, VA, United States; ^3^Department of Psychiatry, First Hospital/First Clinical Medical College of Shanxi Medical University, Taiyuan, China; ^4^Research Centre for Medical Genetics, Moscow, Russia; ^5^Moscow Institute of Physics and Technology, Dolgoprudny, Russia

**Keywords:** senescence, lung, chronic inflammation, aging, tissue remodeling, network analysis

## Abstract

Pathogenesis of chronic obstructive pulmonary disease (COPD) is dependent on chronic inflammation and is hypothesized to represent organ-specific senescence phenotype. Identification of senescence-associated gene drivers for the development of COPD is warranted. By employing automated pipeline, we have compiled lists of the genes implicated in COPD (*N* = 918) and of the genes changing their activity along with cell senescence (*N* = 262), with a significant (*p* < 7.06e^–60^) overlap between these datasets (*N* = 89). A mega-analysis and a partial mega-analysis were conducted for gene sets linked to senescence but not yet to COPD, in nine independent mRNA expression datasets comprised of tissue samples of COPD cases (*N* = 171) and controls (*N* = 256). Mega-analysis of expression has identified *CD38* and *TNFRSF12A* (*p* < 2.12e^–8^) as genes not yet explored in a context of senescence–COPD connection. Functional pathway enrichment analysis allowed to generate a model, which explains accelerated aging phenotypes previously observed in COPD patients. Presented results call for investigation of the role of TNFRSF12A/CD38 balance in establishing a vicious cycle of unresolvable tissue remodeling in COPD lungs.

## Introduction

Aging is a fundamental biological process accompanied by changes in the structure and functions of vital organs, including the lungs. Chronic obstructive pulmonary disease (COPD) is associated with persistent airway inflammation and manifests as a steady decline in the lung function, increase in gas trapping, enlargement of the distal air spaces, and the loss of elastic recoil (Vestbo, 2014). According to the 2007–2012 NHANES survey, approximately 5.2% of US adults aged 40–79 were diagnosed with COPD (Tilert et al., 2018).

Current understanding of the pathophysiology of COPD emphasizes pivotal role of chronic inflammation, which is evident from an increase in neutrophils, macrophages, and CD8^+^ T-cell counts throughout the airways, and related release of inflammatory mediators including IL−8, TNF−α, leukotrienes, and reactive oxygen species (ROS) (Barnes, 2014). It is generally accepted that both cellular and molecular pro-inflammatory players act in concert to form an inflammation promoting feedback loop. Some experts, however, suggest that the confinement of the process of inflammation in the airway wall, where the COPD-related remodeling occurs, is debatable (Eapen et al., 2017). Moreover, epidemiological studies point toward a strong systemic component of COPD, with is often accompanied by muscle wasting, cachexia, and cardiovascular involvement (Rubinsztajn et al., 2019). This is one of the reasons why COPD is now considered as an example of organ-specific accelerated aging, with increase in oxidative stress and alteration in the extracellular matrix leading to the development of senescent cell phenotypes (Mercado et al., 2015). Previous gene expression studies produced a list of hundreds of genes, which are both linked to COPD and aging promoting (Brandsma et al., 2017; de Vries et al., 2018), paving a solid foundation to explore the association between COPD and aging at a genetic level. Here, we hypothesize that genes with activity or expression levels increased in aged individuals may also play roles for the etiology of COPD.

To test this hypothesis, we conducted large-scale literature-based disease–gene relationship analysis to compile the lists of genes implicated in COPD and, separately, in senescence, and subtracted the genes previously implicated in both of these phenotypes. For the genes previously identified as senescence-related but not yet highlighted by any COPD studies, a mega-analysis and a partial mega-analysis were completed to evaluate their expression patterns in multiple independently collected COPD-related transcriptomic datasets. Functional pathway enrichment analysis allowed us to generate a model that centers on two novel COPD genes, *CD38* and *TNFRSF12A*, and explains the accelerated aging phenotypes previously observed in COPD patients.

## Materials and Methods

The workflow was organized as follows. First, a large-scale literature mining effort for COPD-related and aging-promoting related gene sets were undertaken in the Pathway Studio environment; these gene sets were compared to identify common genes and age-promoting specific genes. Then, for each gene from the list implicated in aging alone, a mega-analysis, followed by a partial mega-analysis were conducted using nine publicly available COPD expression datasets retrieved from Gene Expression Omnibus (GEO)^1^. For these genes that showed a significant change in expression across analyzed datasets, a Gene Set Enrichment Analysis (GSEA) and literature-based functional pathway analysis were conducted. In addition, possible influences of sample size, population region, and study date on the gene expression levels in COPD were investigated by a multiple linear regression (MLR) model.

### Extraction of Relation Data From Literature

Relation data for genes previously associated with either senescence or COPD were extracted from existing literature in the Pathway Studio environment^2^ and arranged in the database Aging_COPD, hosted at http://database.gousinfo.com. The downloadable form in Excel is available at http://gousinfo.com/database/Data_Genetic/Aging_COPD.xlsx. Beside the list of analyzed genes (Aging_COPD→Aging_alone genes, Aging_COPD→COPD_alone genes, and Aging_COPD→Common genes), supporting references for each disease-gene relation were retrieved (Aging_COPD→Ref for Aging_alone genes, Aging_COPD→Ref for COPD_alone genes, and Aging_COPD→Ref for Common genes) to include both titles of respective papers and particular sentences describing identified relationships. The database allows automated mining for supportive statements underlining the association of each candidate gene with senescence and/or with COPD.

### Selection of Gene Expression Datasets

To compile the list of gene expression datasets, publicly available, the GEO database was searched using the keyword “chronic obstructive pulmonary disease,” which has returned 171 entries. Datasets were extracted with no selection bias and covered the entire GEO contents. The following standards were applied for the further filtering: (1) The organism is *Homo sapiens*; (2) The data type is RNA expression; (3) The sample size is no less than 10; and (4) the study design is case control. Finally, a total of nine datasets remained available for the mega-analysis of expression patterns (Table 1). For each dataset, raw data files rather than the reported research outputs were used to perform the analysis.

**TABLE 1:** Datasets utilized for chronic obstructive pulmonary disease (COPD)-senescence expression mega-analysis.

Study name	Dataset GEO ID	*n* Control	*n* Case	Country	Tissue
Bastos et al., 2016	GSE37768	20	18	Spain	Peripheral lung
Kalko et al., 2014	GSE27543	6	10	United Kingdom	Musculus vastus lateralis
Kalko et al., 2013	GSE27536	24	30	United Kingdom	Musculus vastus lateralis
Ezzie et al., 2012	GSE38974	9	23	United States	Lung
Tilley et al., 2011	GSE11784	135	22	United States	Airway epithelial cells
Poliska et al., 2011	GSE16972	6	6	Hungary	Alveolar macrophage; peripheral blood monocytes
Bosco et al., 2010	GSE19903	10	10	Australia	Induced sputum cells
Boelens et al., 2009	GSE12472	27	36	Netherlands	Large bronchial
Bhattacharya et al., 2008	GSE8581	19	16	United States	Lung

### Mega-Analysis and Partial Mega-Analysis of Expression Datasets

The expression data were normalized and log2-transformed. Mega-analysis allows pooling of individual-level biological endpoint data across datasets by introducing appropriate correction for between-study variations selected by modeling (Seifuddin et al., 2013). Both the fixed-effect model and random-effect model (Borenstein et al., 2010) were employed to study the effect size of senescence-related genes on COPD. For each expression dataset, the log fold change (LFC) was calculated for the COPD samples and used as the index of effect size. Results from both mega-analysis models were compared. In order to study the variance within and between different datasets, the heterogeneity of the mega-analysis was assessed. In case when total variance *Q* was equal to, or smaller than, the expected between-study variance df, the statistic ISq = 100% × (*Q* – df)/*Q* was set to 0, and a fixed-effect model was selected for the mega-analysis. Otherwise, a random-effect model was selected. The *Q*–*p* represents the probability that the total variance is explained by within-study variance only.

To discover the genes significantly altered in some, but not all studied datasets, we performed a partial mega-analysis, where the top 50% datasets were employed for the mega-analysis of each gene. The “Top datasets” were defined for each gene individually as datasets demonstrating larger absolute value of effect size than the rest of the datasets. Analyses were conducted using MATLAB (R2017a) mega-analysis package.

Results from both mega-analysis and partial mega-analysis were compared to identify significant genes according to the following the criteria, *p* < 1.00e^–7^ and effect size (LFC) > 0.49 or < −0.74. When a gene presented an effect size LFC > 0.49 or < −0.74 in the mega-analysis, it means that the change in the expression level of the gene had increased by more than 40%, or decreased by more than 40%. While we present all the mega-analysis results in the Aging_COPD→Mega-analysis and Aging_COPD→Partial-Meta, the discussion will be focused on those genes that satisfy the significance criteria outlined above.

### Gene Set Enrichment Analysis and Shortest Path Analysis

To gain functional insights into the set of genes previously described as involved in senescence and in COPD and, separately, the set of genes showing significance in the mega-analysis of senescence-related genes, which were not yet described as COPD contributors, the GSEA has been conducted in the Pathway Studio environment. The GSEA results were reported with enrichment *p*-values corrected using the Bejnamini–Hochberg false discovery ratio (FDR) procedure (Reiner et al., 2003). In addition to the GSEA, for each gene set, a literature-based functional pathway analysis was conducted using the “Shortest Path” module of the Pathway Studio^3^.

### Multiple Linear Regression Analysis

A MLR analysis was employed to study the possible influence of the following three factors on the gene expression changes: sample size, population region, and study date. Values of *p* and 95% confidence interval (CI) were reported for each of these factors. The analysis was performed in MATLAB (R 2017a) with the “regress” statistical analysis package.

## Results

### Many Senescence-Associated Genes Are Not yet Studied in COPD Context

Pathway Studio-guided literature data mining for the genes associated with aging and senescence has yielded 262 genes, while COPD phenotypes were associated with 918 genes. Despite a significant overlap between aging/senescence-related genes and COPD-related genes (89 genes; *p* = 7.06e^–60^), over half of the set of aging/senescence-related genes (*N* = 173 or 66.03%) have not been previously implicated in COPD. A complete list of these 173 aging/senescence-related genes along with support evidence and references is presented in the following files: Aging_COPD→Aging_alone genes and Ref for Aging_alone genes.

### Mega- and Partial Mega-Analysis of Gene Expression Highlights TNFRSF12A and CD38

Only two genes, *TNFRSF12A* and *CD38*, have satisfied the significance criteria outlined in Table 2. Specifically, for an increased expression of *TNFRSF12A*, both mega-analysis and partial-meta analysis results were positive, while *CD38* was identified as significantly involved by the partial-meta analysis only.

**TABLE 2:** Novel aging/senescence-related genes as highlighted by mega-analysis (MA) and partial MA expression.

Gene/type of analysis	Significance	Random-effect model	# Study	LFC	*p*-value	ISq (%)	*p*-value–Q
TNFRSF12A/partial MA	Yes	NO	4	0.54	5.80e^–10^	0	1.00
TNFRSF12A/MA	Yes	NO	9	0.51	1.53e^–9^	0	0.96
CD38/partial MA	Yes	NO	4	-0.89	2.12e^–8^	0	0.82
CD38/MA	No	Yes	8	-0.49	2.19e^–2^	17.34	0.29

*LFC, log fold change (the effect size); *p*-value represents the probability that the fold change is equal to 0; ISq = 100% × (*Q* – df)/Q represents the percentage of between-variance over total variance; *p*-value–Q represents the probability that the variance is explained by within-study variance only.*

**FIGURE 1:**
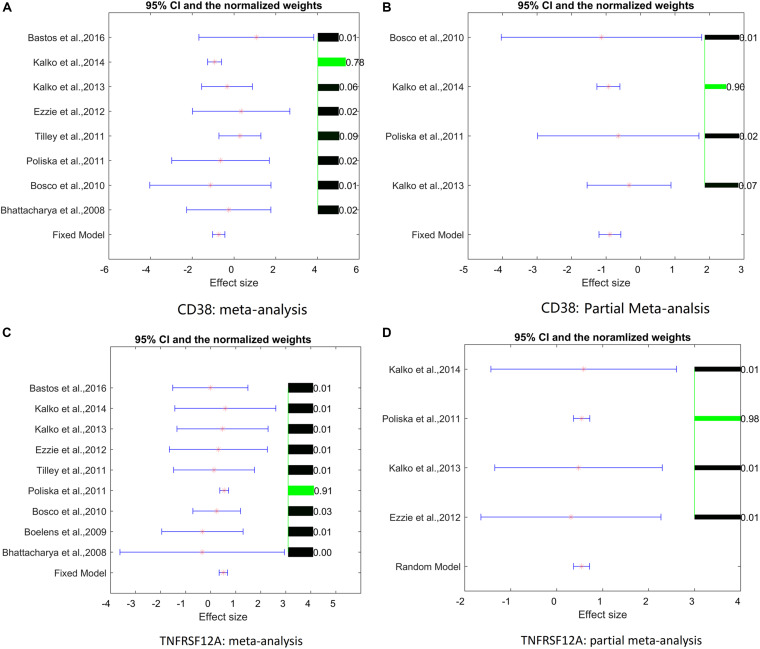
Effect sizes, 95% confidence intervals, and weights for each of two genes. **(A)** Mega-analysis results for *CD38*. **(B)** Partial mega-analysis results for *CD38*. **(C)** Mega-analysis results for *TNFRSF12A*. **(D)** Partial mega-analysis results for *TNFRSF12A*. The bar plot on the right of each figure represents the normalized weights for each dataset/study, ranged within (0, 1); the brighter (green) the color, the larger the relative weight of the study as labeled right next to the bar. For each dataset, the star (in red) and lines (in blue) on the left are the mean of effect size (log fold change), and 95% confidence interval (CI), respectively. References for datasets could be traced by their Gene Expression Omnibus (GEO) numbers.

Analysis of study heterogeneity showed that, for *TNFRSF12A*, there was no significant between-datasets variance for both mega-analysis and partial mega-analysis (ISq = 0, *p*–*Q* > 0.96), and therefore, a fixed-effect model was selected for this gene. For *CD38*, in mega-analysis, the between-datasets variance had accounted for 17.34% of the overall variance. When the random-effect model was used, outputs were not significant. Therefore, for *CD38*, only partial mega-analysis results were employed. For each gene, effect sizes, 95% confidence intervals, and weights of each study are presented in Figure 1.

**FIGURE 2:**
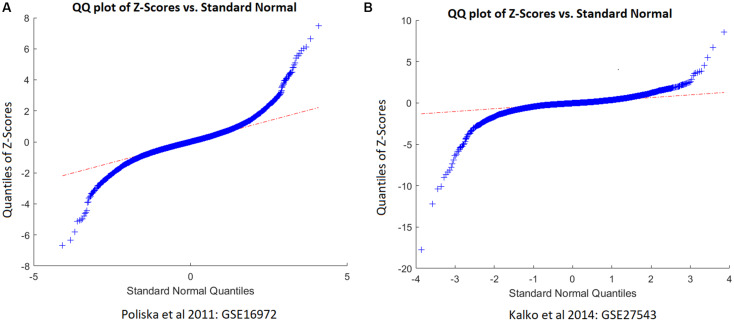
QQ plot of the z-scores of chronic obstructive pulmonary disease (COPD) patients versus standard normal distribution. **(A)** QQ plot built for dataset GSE16972. **(B)** QQ plot built for dataset GSE27543.

As shown in Figure 1, two studies/datasets – GSE16972 for *TNFRSF12A* and GSE27543 for *CD38 –* demonstrated relatively small variances of effect size, leading to high *z*-scores and high weights within the mega-analysis. To determine whether these small variances were specific for the two genes, or were due to the peculiarities of data distribution within the datasets, we studied the distribution of the z-scores for all the genes differentially expressed in COPD patients suing empirical quantile–quantile plots (QQ plot), against the normal distribution, as shown in Figure 2. Even if the *z*-scores of these two datasets were not well fit to a normal distribution, their overall distribution was not that wide, with most of the genes demonstrating near-zero values. Therefore, the small variance corresponding to high *z*-scores observed for the *TNFRSF12A* and *CD38* genes was not different to distribution expression values for other genes, not highlighted by mega-analysis.

### Multiple Linear Regression Analysis

A MLR analysis was conducted to explore the potential influence of three parameters on the expression levels of the genes in the case of COPD. Analysis of the MLR models shows that the region, where each study was performed, exerted significant influence on the expression fold changes detected for *TNFRSF12A* and *CD38* genes with *p* < 3.10e^–4^ and 2.30 < e^–3^, respectively, nor were sample sizes not studying publishing dates were significant influencers.

### GSEA Results

Input list for GSEA analysis is located in Aging_COPD→GSEA. In GSEA analysis of 91 genes implicated both in COPD and in senescence phenotypes (previously described genes, *N* = 89 genes; genes discovered in this study by mega-analysis, *N* = 2), a total of 113 GO terms were significantly enriched (*p* < 1e^–10^). Table 3 presents the Top 10 GO terms with enrichment values of *p* < 1.19e^–21^. Noteworthy, *TNFRSF12A* has been included in 9 of 113 enriched pathways, and in 3 of the Top 10 pathways (Table 3). *CD38* was a part of 26 of the 113 enriched pathways, including in 4 of the top 10 pathways (Table 3). One of the GO terms, Apoptotic Signaling Pathway, retrieved a gene list with both *TNFRSF12A* and *CD38*.

**TABLE 3:** Top 10 GO terms enriched by 91 genes linked to both senescence/aging and COPD phenotypes.

GO ID	GO name	# of entities	Overlap	*p*-value	Novel gene included
0070482	Response to oxygen levels	544	33	4.4E^–27^	*CD38*
0010942	Positive regulation of cell death	870	37	1.14E^–25^	*TNFRSF12A*
0001666	Response to hypoxia	424	29	4.49E^–25^	*CD38*
0036293	Response to decreased oxygen levels	461	29	3.94E^–24^	*CD38*
0048545	Response to steroid hormone	418	27	1.21E^–22^	*CD38*
0031960	Response to corticosteroid	325	25	1.22E^–22^	No
0043068	Positive regulation of programmed cell death	800	33	3.06E^–22^	*TNFRSF12A*
0051384	Response to glucocorticoid	299	24	4.27E^–22^	No
0019221	Cytokine-mediated signaling pathway	676	31	4.81E^–22^	*TNFRSF12A*
0009636	Response to toxic substance	634	30	1.19E^–21^	No

### Pathway Studio-Guided Analysis of Existing Literature

**FIGURE 3:**
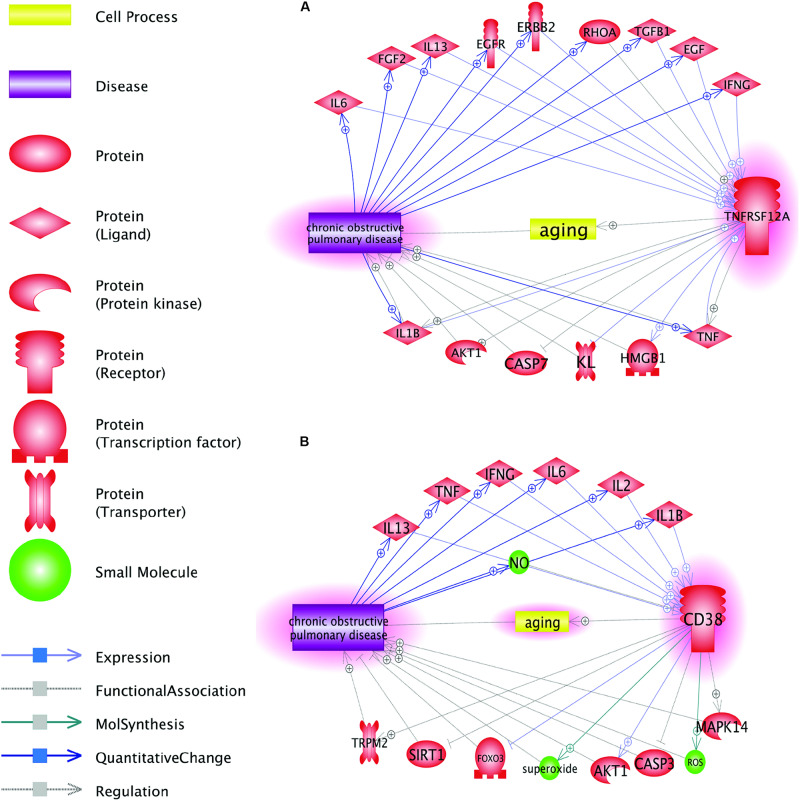
Function pathway analysis between COPD and the two genes: CD38 and TNFRSF12A. This network was generated in Pathway Studio environment (www.pathwaystudio.com). Each relation (edge) in the figure has one or more supporting references. **(A)** Pathways connecting COPD and gene TNFRSF12A; **(B)** Pathways connecting COPD and gene CD38.

As COPD-related genes, *TNFRSF12A* and *CD38*, were identified *de novo*, after specific exclusion of the genes previously described as involved in COPD, gene-specific PubMed search confirmed that neither *TNFRSF12A* nor *CD38* were previously discussed in the mechanistic context of COPD. However, the Shortest Path analysis performed in the Pathway Studio data mining environment revealed a number of plausible connections between these two genes and COPD, with a set of common interactions (Figure 3).

Figure 3A shows that *TNFRSF12A* may influence the pathogenesis of COPD through multiple pathways. As an example, let us trace the connection *TNFRSF12A*→HMGB1→COPD. High-mobility group box-1 (HMGB1) modulates the balance between senescence and apoptosis in response to genotoxic stress, with higher expression levels of this protein profoundly shifting the balance toward senescence (Lee et al., 2019). Increased TWEAK signaling through larger amounts of available TNFRSF12A receptor potently induces HMGB1 expression and secretion (Moreno et al., 2013). Increases in extracellular concentrations of HMGB1 are proportional to the severity of COPD itself (Hou et al., 2011). Outside the cells, HMGB1 associates with numerous other proteins and signals back to the cell through the receptor for advanced glycation end products (RAGE) and toll-like receptor-4 (TLR-4), thus behaving as a typical damage-associated molecular pattern (DAMP) (Wong et al., 2018; Paudel et al., 2019). The details for all other relations presented in Figure 3 are described in Aging_COPD→Pathway Analysis. This reference information includes the types of the relationship, amount of underlying supporting references, and related sentences where these relationships have been identified and described. Evidence presented in Figure 3 also indicates that perpetuated overexpression of *TNFRSF12A* may serve one of the drivers for a vicious circle that keeps COPD patients from resolving inflammation in their lungs.

## Discussion

We performed this study in an attempt to identify novel, not yet described molecular pathways, which link the process of tissue and cell aging and the development of COPD. By removing all known intersections between curated gene sets involved in each of these pathophysiological processes, we ensured that uncovered senescence-related contributors to COPD had not been already described as such. Through the Pathway Studio-guided literature mining, a total of 173 genes involved in the senescence, but not in COPD, were discovered. These genes were investigated for consistent evidence of the changes in their expression in COPD phenotypes across nine mRNA expression datasets acquired from GEO (Table 1).

In cross-dataset mega-analysis of gene-level expression patterns, expression levels of seven senescence-related genes were significantly altered in COPD compared to normal lungs (*p* < 0.05, see in Aging_COPD→Meta_Analysis). When across dataset thresholds were lowered by applying partial mega-analysis techniques, a total of 18 senescence-related genes were highlighted as possibly involved in COPD (see in Aging_COPD→Partial Meta). However, only two senescence-related genes, *TNFRSF12A* and *CD38*, have passed pre-selected criterion of the significance of association, which were set at *p* < 1e^–7^ and LFC of >0.49 or <−0.74 (Table 2 and Figure 2). In particular, in meta- and partial meta-analyses, LFCs of observed *TNFRSF12A* expression levels were at 0.51 and at 0.54, respectively, demonstrating that expression levels of *TNFRSF12A* in COPD samples were consistently increased by more than 40%. When similar criteria were applied to *CD38*, its expression levels were found to be decreased in COPD samples by more than 40% (Table 2), with the only difference that the changes in *CD38* expression levels have passed the significance criteria in partial mega-analysis only.

For both molecules, their roles in the senescence and aging-associated diseases are well described, while no apparent connections to COPD have been reported so far. *TNFRSF12A* encodes for an exclusive receptor for tumor necrosis factor-related weak inducer of apoptosis (TWEAK); jointly, this interacting pair of molecules is involved in age-associated pathological changes in skeletal muscle and other organs (Van Kirk et al., 2011; Tajrishi et al., 2014; Hénaut et al., 2016). CD38 is one of the main NAD-degrading enzymes, which increases its expression in aging tissues and is directly responsible to age-related NAD decline (Horenstein et al., 2013; Camacho-Pereira et al., 2016). When the levels of NAD+ are low, a senescence-associated secretory phenotype (SASP) to a certain degree diminishes its damaging power as a part of a fundamental trade-off between aging and energy available to the cells (Mendelsohn and Larrick, 2019). Moreover, adenosine, which is produced in CD38-dependent reaction, further suppresses local immune response (Horenstein et al., 2013). In healthy aging, SASP cytokines promote an expression of CD38 (Chini et al., 2019). In case of lungs affected by COPD, a decrease in CD38 expression was noted in at least some datasets. It should be noted, however, that some COPD datasets do not comply, possibly due to the stage-specific differences in the SASP response of COPD lungs (Parikh et al., 2019). As senescent cells are also capable of inducing surface CD38 expression on macrophages and endothelial cells (Chini et al., 2019), particular balance between CD38-lowering and CD38-enhancing forces may be dependent upon tissue composition and/or disease stage of collected samples and, therefore, may be dataset specific.

Notably, a number of previous studies connected COPD to an increased risk of developing non-small cell lung carcinoma (NSCLC), irrespective of the pack-years history of their smoking (Houghton, 2013; Ng KeeKwong et al., 2017). Both *TNFRSF12A/TWEAK* pair (Whitsett et al., 2014) and CD38, along with its interaction partner CD31 (Malavasi et al., 2008) are involved in multiple cancers, including NSCLC, where they contribute to escape from PD-1/PD-L1 blockade (Chen et al., 2018; Konen et al., 2019) and cytotoxic therapy (Whitsett et al., 2014). Obvious importance of *TNFRSF12A/TWEAK* molecules for the survival of cancers cells prompted the development of the respective targeting agents, which already met initial success in clinical and pre-clinical trials [see Hu et al., 2017, for review]. Our findings point at the potential applicability of the abovementioned therapeutics in COPD context.

The results of the GSEA analysis support the association between senescence and COPD, with 113 pathways uncovered as significantly enriched and connected to both of these phenotypes (*p* < 1e^–10^; see Table 3 and Aging_COPD→GSEA). Among them, we have identified 9 and 26 significantly enriched pathways, where either *TNFRSF12A* or *CD38* were listed as shared genes, respectively. Notably, *CD38* plays a role in many enriched oxygen/hypoxia and steroid hormone-related pathways, which both have been linked to COPD previously (Marwick et al., 2013; Wada and Takizawa, 2013). On the other hand, a number of enriched cytokine-mediated signaling pathways, positively regulating cell apoptosis and influencing angiogenesis, include TNFRSF12A as their integral part; these pathways have also been implicated in COPD pathogenesis (Vanella et al., 2017; Kropski et al., 2018).

Employing Pathway Studio-guided “Shortest Path analysis” allowed us to explore plausible connections between these two genes and COPD, simultaneously highlighting a set of common interactors, which includes the signaling molecule and the pro-inflammatory cytokines. Among these, a connection of both TNFRSF12A and CD38 proteins to AKT1 is remarkable, as the stimulation of IGF1/AKT1 axis via the targeting of p16-induced senescence was recently shown to alleviate at least some phenotypic features of COPD (Cottage et al., 2019). Notably, among other connecting molecules are pro-inflammatory cytokines TNF-α, IL-1β, IFN-γ, and IL-6, which are the part of the SASP phenotype (Barnes, 2014; Ohtani, 2019).

Importantly, this study is not free of limitation. In particular, we acknowledge that the study pipeline was executed *in silico*, with no experimental validation of the findings, and that the study set was relatively small, in part, due to the strict quality control of selected datasets. Nevertheless, we believe that the overall conclusion of our study may further our understanding of COPD.

**FIGURE 4:**
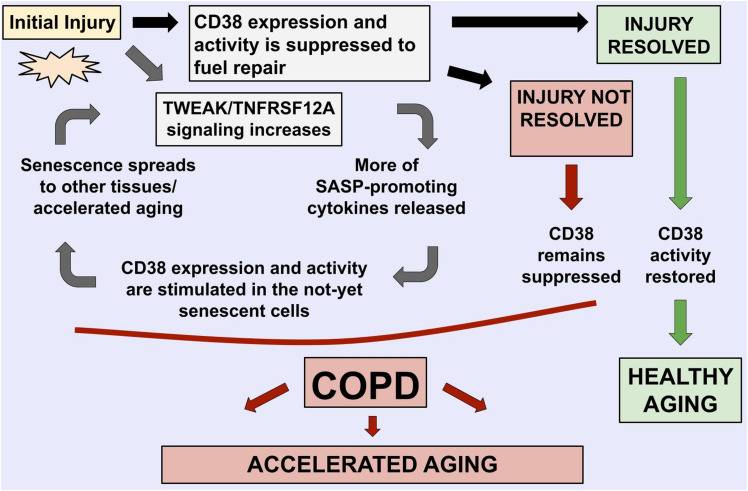
Mechanistic model highlights the roles of *TNFRSF12A* and *CD38* in COPD-related accelerated aging phenotypes, with their balance defining if the lung injury is resolved.

In particular, we noted that CD38/TNFRSF12A and COPD relationships demonstrate the features of bidirectional, self-propagating cycle rather than a unidirectional regulation pathway (Figure 4). We speculate that, in COPD, stimulation of TWEAK/TNFRSF12A signaling enhances tissue remodeling by stimulation migration of the cells and the SASP cytokine production [these statements are supported by experiments described in Refs. (Wang et al., 2017; Das et al., 2018; Liu et al., 2019)], which, in turn, induce CD38 mRNA and protein expression, and stimulate activity of this NADase in adjacent non-senescent cells, including populations of macrophages and endothelia [supported by experiments described in Parikh et al., 2019], with the resultant depletion of NAD+ contributing to accelerated aging observed in COPD patients (Triest et al., 2019), and to further increase in TWEAK/TNFRSF12A signaling. The resultant model is described in Figure 4, with relative levels of CD38/TWEAK expression establishing whether lung inflammation would resolve or would form a vicious cycle of COPD.

## Conclusion

Using a large-scale literature data mining and gene-level mega-analysis of multiple gene expression datasets, we specifically searched for novel senescence-associated genes, which may also contribute to COPD. We show that genes *TNFRSF12A* and *CD38* encode factors previously unrecognized as COPD contributors, and have generated the model that explains accelerated aging phenotypes previously observed in COPD patients, and calls for investigation of the balance of TNFRSF12A/CD38 proteins as the key to establishing vicious cycle of unresolvable tissue remodeling in COPD lungs.

## Data Availability Statement

The datasets analyzed in this study can be found in the GEO repository (https://www.ncbi.nlm.nih.gov/geo/). Relation data for genes previously associated either with senescence or with COPD were extracted from existing literature in the Pathway Studio environment (www.pathwaystudio.com) and arranged in the database Aging_COPD, hosted at http://database.gousinfo.com. The downloadable form in Excel is available at http://gousinfo.com/database/Data_Genetic/Aging_COPD.xlsx.

## Author Contributions

YD, RC, and HC contributed to the conception and design of the study. HC organized the database and performed the Pathway Studio work. RC and YD retrieved the expression data and performed the statistical analysis. RC and HC wrote the first draft of the manuscript. AB provided the interpretation of the data and built the COPD model. AB and HC wrote the final version of the manuscript. All authors contributed to the manuscript revision, read, and approved the submitted version.

## Conflict of Interest

The authors declare that the research was conducted in the absence of any commercial or financial relationships that could be construed as a potential conflict of interest.

## Footnotes

1 https://www.ncbi.nlm.nih.gov/geo/

2 www.pathwaystudio.com

3 http://www.pathwaystudio.com
